# Should Genetic Testing for Cancer Predisposition Be Standard-of-Care for Women with Invasive Breast Cancer? The Murtha Cancer Center Experience

**DOI:** 10.3390/cancers12010234

**Published:** 2020-01-17

**Authors:** Seth K. Rummel, Leann A. Lovejoy, Clesson E. Turner, Craig D. Shriver, Rachel E. Ellsworth

**Affiliations:** 1 Chan Soon-Shiong Institute of Molecular Medicine at Windber, 620 Seventh Street, Windber, PA 15963, USA; s.rummel@wriwindber.org (S.K.R.); l.lovejoy@wriwindber.org (L.A.L.); 2 Uniformed Services University of the Health Sciences, 4301 Jones Bridge Road, Bethesda, MD 20814, USA; clesson.turner@usuhs.edu (C.E.T.); craig.shriver@usuhs.edu (C.D.S.); 3 Clinical Breast Care Project, Murtha Cancer Center Research Program, 4494 North Palmer Road, Bethesda, MD 20889, USA; 4 Henry M. Jackson Foundation for the Advancement of Military Medicine, 6720A Rockledge Drive, Bethesda, MD 20817, USA

**Keywords:** breast cancer, genetic testing, BRCA1/BRCA2, standard-of-care

## Abstract

Currently, genetic testing is offered only to women diagnosed with breast cancer who meet a defined set of criteria and is not included as standard-of-care treatment at the time of diagnosis. Thus, a significant number of women diagnosed with breast cancer may miss the opportunity for precision medical treatment and risk management. The effects of eligibility, timing, and uptake of genetic testing were evaluated in a cohort of women with invasive breast cancer diagnosed between 2001–2018. Risk status was estimated using NCCN *BRCA1/2* testing criteria and panel testing was performed for all women who had genomic DNA available. Of the 1231 women, 57.8% were eligible for genetic testing. Uptake of testing within high-risk women was 42.7% of which 6.6% pursued clinical testing only after a second tumor event. Mutation frequencies were 15.8%, 5.5%, and 4.0% in high-risk women with clinical testing, high-risk women without clinical testing, and low-risk women, respectively. More than 4% of all patients harbored pathogenic or likely pathogenic mutations detected only in the research setting. Inclusion of panel testing at the time of diagnosis would allow for appropriate surveillance and treatment strategies to be employed to reduce the risk of secondary tumors and improve patient outcome.

## 1. Introduction

Discovery of the breast cancer 1 (*BRCA1*) and breast cancer 2 (*BRCA2*) genes [1,2] led to the development of genetic tests to identify individuals at risk for hereditary breast and ovarian cancer (HBOC). Early genetic testing involved costly and time consuming sequencing of each gene in a largely sequential manner. Two decades after the BRCA genes were identified, technical advances in next generation sequencing allowed for the simultaneous assessment of multiple genes, decreasing cost and time to return of test results. In conjunction, the Supreme Court of the United States overturned Myriad Genetics’ patent on the *BRCA1* and *BRCA2* genes, which allowed for the development of multi-gene cancer predisposition panels that today are offered by multiple commercial companies [3].

As technologies to identify mutations in cancer predisposition genes have evolved, the utility for identifying patients with hereditary cancers has expanded from personal or family risk assessment to personalized treatment strategies. For example, women with *BRCA1* or *BRCA2* mutations may benefit from double mastectomy to reduce risk of contralateral disease [4] and demonstrate improved response to platinum agents and poly(ADP-ribose) polymerase (PARP) inhibitors [5,6,7,8]. For patients with germline mutations in *ATM*, *CHEK2*, *NBN*, *NF1*, and *PALB2*, enhanced surveillance through addition of MRI may be warranted while breast cancer patients with mutations in *BRIP1* or mismatch repair genes may benefit from risk-reducing salpingo-oophorectomy (RRSO), endoscopy, or colonoscopy.

Despite the clinical benefits of identifying germline mutations in cancer predisposition genes, genetic testing is not currently offered to all women with breast cancer. When first offered in 1996, clinical testing was reserved for women diagnosed at an early age or with a significant family history of breast and ovarian cancer [9,10]. Guidelines from the National Comprehensive Cancer Network (NCCN) have evolved to include an expanded family history of cancers of the prostate and pancreas as well as breast and ovarian, and a diagnosis of triple negative breast cancer at <60 years of age, with or without a family history (https://www.nccn.org/professionals/physician_gls/pdf/genetics_screening.pdf). After over 20 years of restricting genetic testing to those women who meet a certain set of criteria, in February 2019, the American Society of Breast Surgeons (ASBS), recognizing that a significant number of test-ineligible women in fact harbor germline mutations in cancer predisposition genes, recommended that all women newly diagnosed or with a personal history of breast cancer should be offered genetic testing to improve patient treatment and provide personal and family risk management strategies (https://www.breastsurgeons.org/docs/statements/Consensus-Guideline-on-Genetic-Testing-for-Hereditary-Breast-Cancer.pdf).

If implemented, these recommendations would afford all breast cancer patients the opportunity for genetic testing; however, testing has not been standardized and the uptake and timing of testing and choice of genes to evaluate may differ significantly between patients. Incorporating panel testing into standard-of-care at the time of diagnosis may improve the identification of ostensibly low-risk patients who harbor germline mutations in cancer predisposition genes, as well as to prevent additional breast tumors or cancers at secondary sites in the patient or family members. To explore the utility of panel testing in all breast cancer patients at the time of diagnosis, a panel of cancer predisposition genes was sequenced in women diagnosed at the Murtha Cancer Center, Walter Reed National Military Medical Center (MCC/WRNMMC) representing three groups: (1) Those who met NCCN guidelines and underwent clinical testing; (2) those who met NCCN guidelines but did not pursue clinical testing; and (3) patients who were ineligible for genetic testing using NCCN criteria.

## 2. Results

Between 2001 and 2018, 1231 females diagnosed with invasive breast cancer at the MCC/WRNMMC enrolled in the Clinical Breast Care Project (CBCP). Seventy-six (6.2%) women were diagnosed with non-breast cancers before their breast cancer diagnosis. One thousand (81.2%) women had at least one first or second degree relative diagnosed with a cancer other than basal cell or squamous cell carcinoma of the skin.

Based on guidelines at the time of their diagnosis, 542 (44.0%) patients were eligible for genetic testing. An additional 170 (13.8%) women would be eligible for testing using version 1.2018 guidelines. Using the NCCN version 1.2018 criteria, high-risk patients were significantly (*p* < 0.001) younger at diagnosis (52.5 years of age), more likely to have a family history of cancer (91.6%), and more likely to have triple negative breast cancer (TNBC) (19.8%) than low-risk women (63.8 years of age, 67.1%, and 6.4%, respectively; Table 1).

Uptake of genetic testing within the high-risk group was 42.7% and was significantly higher (*p* < 0.001) among women eligible for testing at diagnosis (281/542, 51.8%) compared to those whose status changed from low to high-risk through changes in NCCN criteria or additional cancer events within the family (23/170, 13.5%). Demographic and clinical data from high-risk women are shown in Table 2. Those who pursued genetic testing were significantly (*p* < 0.001) younger and more likely to be college educated.

Time-to-testing ranged from time of diagnosis to 15.3 years post-diagnosis. The mean time-to-testing was significantly (*p* < 0.001) shorter in women who were eligible for testing at diagnosis (0.72 years) compared to those women whose status changed from low to high-risk (5.38 years). Twenty (6.6%) women had testing only after a second tumor event (19 breast, one ovary).

Clinical testing (*n* = 304) was performed using the Ashkenazi 3-site mutation panel (*n* = 5), *BRCA1/2* sequencing (*n* = 142), Lynch syndrome testing (*n* = 1), and multi-gene panel testing (*n* = 156). Of the patients who had their original testing limited to *BRCA1* and/or *BRCA2*, eight BRCA negative patients later underwent additional panel testing. One woman was diagnosed with a pathogenic *TP53* mutation after an ipsilateral recurrence, two years after her original breast cancer diagnosis.

Forty-eight (15.8%) of the women with clinical testing carried a pathogenic or likely pathogenic mutation (Table 3). Within the 111 women with limited (*BRCA1* and/or *BRCA2* only) testing who later had panel testing performed in the research setting, 10 had pathogenic/likely pathogenic mutations in non-BRCA genes. Among 346 high-risk women who did not pursue clinical testing, 19 (5.5%) harbored pathogenic or likely pathogenic mutations detected in the research laboratory. Likewise, in 429 women classified as low-risk, 17 (4.0%) had pathogenic/likely pathogenic mutations, including three women with *BRCA2* mutations. Overall, the frequency of pathogenic or likely pathogenic mutations was 11.8% in high-risk compared to 4.0% in low-risk patients.

Because prophylactic mastectomy has long been recommended for women with BRCA1 and BRCA2 mutations, the effects of delayed or lack of testing were evaluated (Table 4). Patients were classified by time-to-testing of <1 year from diagnosis (*n* = 24), >1 year from diagnosis (*n* = 8) or no clinical testing (*n* = 13). One woman did not have a reported date of testing and was excluded from analysis. The frequency of prophylactic mastectomy was 86% in women who had testing within one year, 63% in those with delayed testing, and 45% in women without clinical results. Breast cancer recurrence or distant metastasis were significantly more likely (*p* < 0.05) in those with delayed testing (50%) or no clinical testing (37%) compared to one (4%) woman who had testing <1 year. In addition, breast cancer survival was significantly lower (*p* = 0.011) in women with delayed testing (25%) compared to those with testing within one year (0%). The breast cancer mortality rate was not significantly higher (*p* = 0.168) in the group of women with only research test results (8%) compared to those who had testing within one year.

In addition to mutations associated with risk of hereditary cancer, pathogenic/likely pathogenic mutations were detected in *ERCC2* (*n* = 1), *FANCA* (*n* = 1), *FANCC* (*n* = 1), *HNF1A* (*n* = 1), *PRF1* (*n* = 1) *RECQL4* (*n* = 2), *WRN* (*n* = 1), and *XPA* (*n* = 1). Associated conditions, such as Xeroderma pigmentosum, Fanconi anemia and Werner Syndrome are all inherited in an autosomal recessive fashion, and each of the individuals in this study carried a single mutant allele. In addition, 49 (17.2%) women who underwent clinical testing harbored at least one variant of uncertain significance VUS, including 12 women with *BRCA1* (*n* = 2) or *BRCA2* (*n* = 10) variants whose pathogenicity has not yet been resolved. Within the patients who had panel testing in the research laboratory, 28.2% harbored at least one VUS, including twelve women with previously unreported variants in *ATM*, *BLM*, *BRCA1*, *BRCA2*, *BRIP1*, and *PALB2*.

## 3. Discussion

During the 25 year period since the *BRCA1* and *BRCA2* genes were identified, a number of additional breast cancer genes have been identified [11]. Management of breast cancer patients who harbor germline mutations in cancer predisposition genes may differ from those patients with sporadic breast cancer. Management ranges from altered surgical and adjuvant approaches for women with *BRCA1* and *BRCA2* mutations, to enhanced surveillance for women with mutations in moderate-penetrance breast cancer genes or in genes associated with risk of other types of cancer [12]. Although criteria have been developed to identify women most likely to harbor germline mutations in cancer predisposition genes, restriction of testing to high-risk patients may exclude a significant number of women who carry pathogenic mutations. For example, application of the NCCN version 2.2017 criteria to a cohort of 1371 newly diagnosed breast cancer patients from Norway who were tested for *BRCA1* and *BRCA2* mutations, revealed that 32 of 38 (88.9%) mutation carriers would have been classified as high-risk [13]. More recently, evaluation of 165,000 high-risk patients found that 5.8% of patients with *BRCA1/2* mutations did not meet NCCN version 1.2018 guidelines [14]. Within our study, 6.5% of patients with *BRCA1/2* mutations were classified as low-risk. These data suggest that stratifying patients into risk groups may miss 5–10% of patients who harbor BRCA mutations.

Expanded testing through multigene panels may be important for identifying significantly more women with hereditary cancers. Our results show that the mutation frequency was 4.1% in *BRCA1* and *BRCA2* and 5.4% in other cancer genes. Similarly, when 35,000 women with breast cancer were tested using a 25 gene panel, approximately 5.2% of the 9.3% of women with pathogenic variants carried mutations in non-BRCA genes [15]. Two large studies have shown that factors associated with mutations in *BRCA1* and *BRCA2*, such as young age, Ashkenazi heritage, family history of breast or ovarian cancer, or having TNBC, were not associated with mutations in non-BRCA genes [15,16]. This suggests that guidelines such as NCCN may exclude from testing a significant number of women with mutations in cancer predisposition genes other than *BRCA1* and *BRCA2*.

The 2019 ASBS statement recommends that all patients with invasive breast cancer be offered genetic testing. Despite these recommendations, testing is not incorporated into standard-of-care. Genetic testing in the United States, even within high-risk women, is underutilized. Recent data from the National Health Interview Survey demonstrated that only 15.3% of high-risk women underwent genetic testing [17]. Uptake of testing was higher within the CBCP/MCC/WRNMMC where 42.7% of test-eligible women pursued genetic testing; however, over half of the eligible patients did not pursue testing. Within the untested high-risk population, 5.5% of women harbored germline mutations in cancer predisposition genes, many of which (>70%) have management guidelines recommended by NCCN. Inclusion of genetic testing into routine patient care may improve the treatment of those with hereditary forms of breast cancer.

Including genetic testing as standard-of-care may also prevent delays in time-to-testing and improve patient treatment by avoiding radiation before later electing for prophylactic mastectomy or utilizing platinum agents or PARP inhibitors for treatment of the primary tumor [18]. Within the nine patients with pathogenic or likely pathogenic mutations who underwent clinical testing more than one year post-diagnosis, one originally had breast conserving surgery with radiation followed by delayed bilateral mastectomy, while five underwent prophylactic mastectomy of the contralateral breast 1–5 years after diagnosis. In addition, four patients recurred before undergoing testing and survival was significantly worse in women with delayed testing. Delays in testing may thus represent a lost opportunity for prevention in these patients.

While incorporating multigene testing into standard-of-care for breast cancer patients may enhance the identification of patients with hereditary forms of breast cancer, universal testing is not without considerations. Although costs for gene sequencing have decreased significantly in recent years, the estimated cost to provide genetic testing to the >260,000 women diagnosed with invasive breast cancer in the United States each year is upwards of $80,000,000 [19]. This figure covers only the cost of sequencing and does not include costs for pre and post-test counseling. In addition, ~60,000 women diagnosed with ductal carcinoma in situ DCIS each year in the United States would also be eligible for testing, further adding to the cost of including germline testing as standard-of-care. In conjunction, there is a shortage of genetic counselors in the United States, thus universal testing would require development of new strategies for delivering test results [20]. In addition to the costs involved, not all mutations detected will be useful in patient management or risk assessment. For example, 0.6% of the patients in this study had pathogenic mutations in *BLM*, which to date, has not been clearly associated with increased risk of breast cancer and no strategies for risk reduction have been developed. Procedures for disclosure of secondary findings, especially those derived from large panel tests, must be determined. Finally, use of multigene panels is associated with increased detection of VUS. The ASBS Consensus Guidelines on Genetic Testing for Hereditary Breast Cancer state that VUS are not clinically actionable and any patient with a VUS should be counseled based on factors such as family history and age at diagnosis [21]. Most importantly is the concept of patient autonomy [22]. If genetic testing were to be incorporated into routine clinical practice, mechanisms must be developed so that the patient may forgo genetic testing.

There are several limitations to this study. Unlike the general population of the United States, all patients in this study had access to comprehensive breast care. Genetic testing services were covered by TriCare insurance, which may account for higher uptake of testing within this cohort (42.7%) as compared to that measured by the National Health Interview Survey (15.3%), as cost has been identified as a significant barrier to genetic testing [23]. This study includes women who voluntarily enrolled in the CBCP, not all women treated within the MCC/WRNMMC system. The high rate of testing in this study may reflect a selection bias of women who were emotionally, mentally, or physically willing to join a research protocol and thus may also be more likely to pursue genetic testing. In addition, rates at which eligible patients declined testing were not available. Offering genetic testing to all patients with invasive breast cancer may reduce physician burdens in identifying and referring eligible patients for testing; however, patient anxiety, education, and cost may diminish test rates and reduce overall impact of democratization of testing. At the variant level, we used a stringent classification system, not including variants with conflicting interpretations or with single submitters. This eliminated 130 additional patients who had variants with weaker levels of classification support, 19 of which harbored variants that had at least one pathogenic or likely pathogenic interpretation. Therefore, the true mutation frequencies may be higher than those reported here. Finally, data was not available for RRSO, chemotherapy, or post-diagnostic mammography and/or MRI. Inclusion of these data in future studies may be useful for determining whether costs associated with expansion of testing at the time of diagnosis alters treatment regimens and surveillance for secondary cancers and whether these measures improve patient outcomes.

## 4. Materials and Methods

### 4.1. Patient Eligibility and Consent

Eligibility criteria for this study required patients to be: (1) At least 18 years of age; (2) mentally competent and willing to sign informed consent documents; and (3) diagnosed with invasive breast cancer at MCC/WRNMMC. All subjects voluntarily agreed to participate in the CBCP and gave written informed consent. Blood samples were collected with approval from the WRNMMC Human Use Committee and Institutional Review Board (protocol WRNMMC IRB #20704).

### 4.2. Clincopathological Data

Individuals with a previous history of stage 0–IV breast cancer were excluded from this study. Family cancer histories through third degree relatives were collected and genetic risk determined using the NCCN *BRCA1/2* testing criteria published from the year of diagnosis as well as version 1.2018 criteria. Genetic test results and date of testing were extracted from the CBCP database for all patients who underwent clinical testing. Triple negative tumors were classified using ASCO/CAP guidelines for determining estrogen receptor, progesterone receptor and HER2 status [24,25].

### 4.3. Multi-Gene Sequencing and Analysis

Genomic DNA was isolated from all patients (*n* = 1043) who had available blood samples using the Gentra Clotspin and Puregene DNA purification kits (Qiagen, Valencia, CA, USA) and quantitated by fluorometry. Libraries were created from 50 ng of DNA using the TruSight Rapid Capture kit and TruSight cancer panel and sequenced on a MiSeq (Illumina, Inc., San Diego, CA, USA) according to manufacturer’s protocols. Data were analyzed using Variant Interpreter (Illumina, Inc., San Diego, CA, USA) and filtered for missense or frameshift mutations, stop gains or losses, initiator codons, in-frame insertions or deletions, and splice site alterations with a minor allele frequency of ≥0.25. The predicted effect of variants was evaluated using the ClinVar database (http://www.clinvar.com/) and classified as pathogenic, likely pathogenic, VUS, likely benign, or benign. Only variants from multiple submitters with no conflicts or that were reviewed by an expert panel were considered pathogenic or likely pathogenic.

## 5. Conclusions

Provision of multigene genetic testing to all breast cancer patients identified an additional 46/1079 (4.3%) women with hereditary cancer beyond the 48 (4.4%) detected through clinical testing. As recommended by ASBS, offering testing to all patients would identify an additional 1.6% of women carrying pathogenic or likely pathogenic mutations currently classified as low-risk. Lifting restrictions on eligibility for genetic testing, however, may not be sufficient to identify the majority of patients with germline mutations in cancer predisposition genes as genetic testing rates—even within an insured system with access to genetic counseling such as MCC/WRNMMC—are below 50%. Within the cohort of high-risk women who did not undergo genetic testing, 2.9% harbored germline mutations. Including testing as standard-of-care at the time of diagnosis may encourage testing among all patients, optimizing the care of and improving outcomes for patients with hereditary cancers.

## Figures and Tables

**Table 1:** Demographic and clinical information for all patients classified as high-risk or low-risk using the National Comprehensive Cancer Network (NCCN) version 1.2018 criteria.

Classification	High-Risk (*n* = 712)		Low-Risk (*n* = 519)		*p*-Value
Age at Diagnosis	52.5 years		63.8 years		<0.001
	N	%	N	%	
Ethnicity					0.167
African American	212	29.8	132	25.4	
Asian American	29	4.1	32	6.2	
Hispanic American	26	3.6	13	2.5	
European American	432	60.7	332	64.0	
Other/unknown	13	1.8	10	1.9	
Personal (non-breast) cancer history					0.478
Yes	41	5.8	35	6.7	
No	671	94.2	484	93.3	
Family history of cancer					<0.001
Yes	652	91.6	348	67.1	
No	60	8.4	171	32.9	
Triple Negative Breast Cancer					<0.001
Yes	141	19.8	33	6.4	
No	553	77.7	479	92.3	
Unknown	18	2.5	7	1.3	

**Table 2:** Demographic and clinical information for patients classified as high-risk at the time of diagnosis.

Classification	Tested (*n* = 304)		Not Tested (*n* = 408)		*p*-Value
Age at Diagnosis	46.8 years		56.3 years		<0.001
	N	%	N	%	
Ethnicity					0.575
African American	87	28.6	125	30.6	
Asian American	14	4.6	15	3.7	
Hispanic American	12	3.9	14	3.4	
European American	188	61.9	244	59.8	
Other/unknown	3	1.0	10	2.5	
Marital status					0.891
Married	235	77.3	321	78.7	
Not married	67	22.0	84	20.6	
Unknown	2	0.7	3	0.7	
Education					<0.001
<College degree	89	29.3	168	41.2	
≥College degree	171	56.3	165	40.4	
Unknown	44	14.4	75	18.4	
Family History					0.990
Yes	229	75.3	308	75.5	
No	73	24.0	97	23.8	
Unknown	2	0.7	3	0.7	
TNBC					0.672
Yes	74	24.3	97	23.8	
No	224	73.7	299	73.3	
Unknown	6	2.0	12	2.9	

**Table 3:** Frequency of pathogenic or likely pathogenic mutations in cancer predisposition genes by risk and testing groups.

Gene	High-Risk Clinical Testing (*n* = 304)		High-Risk BRCA Negative with Panel Testing (*n* = 111)		High-Risk Research Results (*n* = 346)		Low-Risk Research Results (*n* = 429)	
	N	%	N	%	N	%	N	%
*BRCA1/2* genes								
*BRCA1*	17	5.6%	0	0.0%	6	1.7%	0	0.0%
*BRCA2*	16	5.3%	0	0.0%	4	1.2%	3	0.7%
Other breast cancer genes								
*ATM*	5	1.6%	1	0.9%	0	0.0%	2	0.5%
*CHEK2*	1	0.3%	0	0.0%	3	0.9%	1	0.2%
*NBN*	2	0.7%	1	0.9%	0	0.0%	1	0.2%
*PALB2*	1	0.3%	1	0.9%	0	0.0%	0	0.0%
*TP53*	4	1.3%	0	0.0%	1	0.3%	0	0.0%
Lynch syndrome genes								
*MSH2*	1	0.3%	0	0.0%	0	0.0%	0	0.0%
Other cancer genes								
*BLM*	0	0.0%	2	1.8%	2	0.6%	2	0.5%
*CDKN2A*	1	0.3%	0	0.0%	0	0.0%	0	0.0%
*FH*	0	0.0%	0	0.0%	0	0.0%	2	0.5%
*MUTYH*	0	0.0%	4	3.6%	3	0.9%	4	0.9%
*NF1*	0	0.0%	1	0.9%	0	0.0%	0	0.0%
*RET*	0	0.0%	0	0.0%	0	0.0%	1	0.2%
*SDHB*	0	0.0%	0	0.0%	0	0.0%	1	0.2%

**Table 4:** Effects on surgical decision making and outcome in 45 women with BRCA1 or BRCA2 mutations by time-to-testing.

Time to BRCA Testing	RRPM ^a^	Time to RRM (Years)	Second Cancer Event	Time to Second Cancer (Years)	Patient Status ^c^ (Years)
Clinical testing <1 year from diagnosis					
	Yes	0.0			NED (0.5)
	Yes	0.0			NED (9.6)
	Yes	0.0			NED (5.2)
	Yes	0.0			NED (4.2)
	Yes	0.0			NED (5.0)
	No				NED (1.9)
	Yes	0.0	DM ^d^	1.9	NED (1.9)
	Yes	0.0			NED (0.1)
	Yes	0.0			NED (2.0)
	NA ^b^				NED (4.8)
	Yes	0.0			NED (1.1)
	Yes	0.0			NED (1.9)
	No				NED (10.0)
	Yes	0.9			NED (2.4)
	Yes	0.0			NED (3.2)
	NA				NED (6.4)
	Yes	1.4			NED (4.4)
	Yes	0.0			NED (3.8)
	Yes	0.0			NED (6.6)
	Yes	0.0			NED (9.4)
	Yes	0.4			NED (14.5)
	Yes	0.0			NED (0.5)
	Yes	0.0			NED (5.1)
	No				NED (9.8)
Clinical testing ≤1 year from diagnosis					
	Yes	1.5			NED (1.5)
	No		Contralateral	5.8	DOD (7.5)
	Yes	2.6			NED (1.4)
	Yes	0.0			NED (3.8)
	No		Contralateral	4.2	DOD (6.41)
	No				NED (8.2)
	Yes	7.9	Ipsilateral	8.2	NED (12.9)
	Yes	10.9	Ipsilateral	10.9	NED (10.9)
Research testing only					
	Yes	0.0	Ipsilateral	3.2	NED (4.9)
	No		Contralateral	11.2	NED (11.7)
	No				NED (8.2)
	Yes	8.9			NED (13.6)
	NA				NED (10.5)
	No		Contralateral	2.8	NED (2.9)
	No				NED (8.7)
	Yes	0.0			NED (8.0)
	No				NED (8.6)
	Yes	0.0			NED (10.6)
	No				DOC (1.1)
	Yes	0.00	DM	1.8	DOD (2.5)
	NA				NED (8.5)

^a^ Risk-reducing prophylactic mastectomy. ^b^ Patient had synchronous bilateral breast cancer and a double mastectomy at the time of diagnosis. ^c^ NED = no evidence of disease, DOC = dead other causes, DOD = dead of disease. ^d^ DM = distant metastasis.
